# Tempo and walking speed with music in the urban context

**DOI:** 10.3389/fpsyg.2014.01361

**Published:** 2014-12-02

**Authors:** Marek Franěk, Leon van Noorden, Lukáš Režný

**Affiliations:** ^1^Faculty of Informatics and Management, University of Hradec KrálovéHradec Králové, Czech Republic; ^2^Institute for Psychoacoustics and Electronic Music, Department of Musicology, Ghent UniversityGhent, Belgium

**Keywords:** walking speed, walking with music, spontaneous synchronization, motivational music, urban walk, auditory bubble, personality

## Abstract

The study explored the effect of music on the temporal aspects of walking behavior in a real outdoor urban setting. First, spontaneous synchronization between the beat of the music and step tempo was explored. The effect of motivational and non-motivational music (Karageorghis et al., 1999) on the walking speed was also studied. Finally, we investigated whether music can mask the effects of visual aspects of the walking route environment, which involve fluctuation of walking speed as a response to particular environmental settings. In two experiments, we asked participants to walk around an urban route that was 1.8 km in length through various environments in the downtown area of Hradec Králové. In Experiment 1, the participants listened to a musical track consisting of world pop music with a clear beat. In Experiment 2, participants were walking either with motivational music, which had a fast tempo and a strong rhythm, or with non-motivational music, which was slower, nice music, but with no strong implication to movement. Musical beat, as well as the sonic character of the music listened to while walking, influenced walking speed but did not lead to precise synchronization. It was found that many subjects did not spontaneously synchronize with the beat of the music at all, and some subjects synchronized only part of the time. The fast, energetic music increases the speed of the walking tempo, while slower, relaxing music makes the walking tempo slower. Further, it was found that listening to music with headphones while walking can mask the influence of the surrounding environment to some extent. Both motivational music and non-motivational music had a larger effect than the world pop music from Experiment 1. Individual differences in responses to the music listened to while walking that were linked to extraversion and neuroticism were also observed. The findings described here could be useful in rhythmic stimulation for enhancing or recovering the features of movement performance.

## Introduction

Recent studies using the experience sampling method documented a variety of environments in which people tend to listen to music. North et al. (2004) reported that roughly half of participants' musical experiences occurred within the home. Listening to music while driving has also become very common. Approximately 18% of musical listening occurred in public spaces, such as restaurants and malls, and on public transport. Finally, approximately 1% of musical listening occurred in the gym/while exercising. The variety of locations where people engaged with music was also documented by Juslin et al. (2008), who showed a roughly similar distribution of musical listening as in the previous study and in the study by Greasley and Lamont (2011). Although the mentioned investigations did not capture their participants listening to music while walking outside, it is known that some people listen to music while walking (Hoffer, 2014). Although there is a body of research on listening to music while driving (e.g., Brodsky, 2001; Wiesenthal et al., 2003; Bull, 2004; Dibben and Williamson, 2007) or on public transport (e.g., Bull, 2001; Simun, 2009; Lyons et al., 2012), only the study by Heye and Lamont (2010) systematically investigated some aspect of the effects of music while walking. The authors were interested in the functions of music listening, emotions elicited by music listening, and awareness of the music and the surroundings. Enjoyment, reducing boredom, creation of emotions, and masking disturbing noises were most common functions of music listening while traveling. These authors also documented that music is often used to create an “auditory bubble” (Bull, 2005), which changes one's perception of the outside environment. People listen to music while traveling in public transport to isolate themselves from other people (Bull, 2001); in this way, they transform urban journeys into private and pleasurable spaces (Simun, 2009). It seems that music listening while walking can have a similar function in some respect, transforming the perception of places as people pass through them.

At present, little is known about the temporal aspect of walking while listening to music. Although few investigations have examined walking while listening to music (e.g., Styns et al., 2007; Leman et al., 2013), there is a relatively large body of research examining the effects of music in various sport activities, including running and treadmill walking. Some authors have explored the influence of music on mood and arousal level before/during sport performance (Nakamura et al., 2010; Lane et al., 2011; Karageorghis and Priest, 2012; Laukka and Quick, 2013), while others studies investigated the effects of listening to synchronous music. Anshel and Marisi (1978) made the first attempt to study the effect of synchronous music by comparing synchronous and asynchronous music conditions using a cycle ergometer endurance task. The results revealed that synchronous music conditions elicited significantly longer endurance than asynchronous music and control conditions. More recently, Simpson and Karageorghis (2006) investigated the effects of synchronous music on 400-m sprint running. Participants completed runs under two music conditions and under a no-music control condition. The authors observed that listening to synchronous music while running elicited faster 400-m sprint performance than the no-music control condition. However, the studies predominantly examined the effects of music on performance improvement, but they did not analyze synchronization processes between tempo of music and tempo of movements.

To date, numerous investigations of synchronization between metronome and finger tapping have been conducted (see Repp, 2005). The recent wide availability of motion capture technology increased the number of studies related to continuous periodic movement, which created the possibility to investigate hand, knee, and whole-body oscillations, visuomotor tracking, eye movements, circle drawing, dancing, as well as walking (see Repp and Su, 2013). The pioneer study by Styns et al. (2007) was focused on the influences of instructed synchronization of walking to music with a broad range of tempi. The authors asked their participants to walk for 45 min on an open-air athletics track while listening to music or metronome. The tempi of the stimuli ranged from 50 to 190 beats per minute (bpm). The data revealed a good response to synchronization in a region between 106 and 130 bmp. The optimal walking tempo for synchronization with music was 120 bpm. Sejdić et al. (2012) showed that the musical track in 15-min long walk led to irregular stride time series in some participants. They reported that the participants walked either to the tempo of the music or that the music caused them to lose concentration on the task. Recently, Mendonça et al. (2014) studied both instructed and spontaneous synchronization with music while walking on a treadmill. The music with the participant's baseline walking frequency, at 5% and 10% above baseline, and at 5% and 10% below baseline was provided. It was found that only the instructed group synchronized with the auditory cues. There is a question, how people relate spontaneously to music while walking in real outdoor environments. Do they spontaneously synchronize with the beat of music?

Another question is how music can influence walking speed or, in general, the speed of movements. To identify differences between the effects of various types of music and to find the optimal type of music for sport performance, Karageorghis et al. (1999) developed a conceptual framework for predicting the motivational qualities of music in exercise and sport environments. The definition of *motivational music* provided by Karageorghis et al. (1999), was borrowed from Gaston's definition of stimulative music (Gaston, 1951, p. 2): “Motivational music tends to have a fast tempo (>120 bpm) and a strong rhythm and is proposed to enhance energy and induce bodily action.” Motivational music stimulates physical activity. Moreover, listening to motivational music would produce psychophysical consequences such as improved mood, reduced perceptions of exertion and changes in arousal (Karageorghis et al., 1999). In contrast, there is music that lacks motivational qualities, which the authors called *oudeterous music* (=neutral in terms of motivational qualities). There is empirical evidence supporting the different effects of motivational and oudeterous music. For example, Karageorghis et al. (2009) studied the effect of motivational and oudeterous music on endurance and a range of psychophysical indices during a treadmill walking task. The results revealed that endurance was increased in both music conditions and that motivational music had a greater ergogenic effect than oudeterous music. These authors concluded that motivational synchronous music can elicit an ergogenic effect and enhance in-task affect during an exhaustive endurance task. More recently, Leman et al. (2013) described a similar concept involving *activating* and *relaxing* music. They studied walking speed while synchronizing with the beat of music in tempo 130 bpm in relation to the sonic features of the music. It turned out that participants listening to activating music while walking took bigger steps, while they took smaller steps while listening relaxing music. Thus, the activating music resulted in faster walking speed, while the relaxing music decreased walking speed. The authors show that the effect of activating and relaxing music does not only depend on tempo but also on the sonic features (e.g., variation in loudness and pitch patterns), which constitute expressive patterns in the music.

Most recently, Moens et al. (in press) demonstrated that walkers can synchronize to the musical beat without being instructed to do so when a special interactive music player is used, which identifies the individual's walking tempo and phase and adapts the music accordingly. It shows the way to the development of an intelligent technological architecture that could deliver flexible rhythmic stimulation adapted to an individual's skills, with the goal of enhancing or recovering features of movement performance. Currently, auditory pacing, a practice, in which subjects perform finger tapping or walking in synchrony with an external auditory stimulus, is used in the rehabilitation of patients with walking difficulties. There are studies which show that auditory pacing stimulation reduces gait variability in patients with Parkinson's disease (e.g., Hayashi et al., 2006; Hausdorff et al., 2007). More recently, Hove et al. (2012) used an interactive device called “Walk-Mate,” which was programmed to carry out phase correction while it asked Parkinson patients to walk.

In contrast to previous studies, our research is intended to examine the temporal aspect of walking with music in naturalistic conditions in a real outdoor urban environment. To study walking with music in a real environment, one must take into account the findings of environmental psychology concerning the relationship between walking behavior and the properties of the surrounding environment. Recently, interest in walking research has grown due to the increase of diseases related to sedentarism (e.g., Brown et al., 2007; Duvall, 2011; Napier et al., 2011). The walkability of a given route is in the focus of interest, which typically involved various variables such as traffic safety, accessibility, pleasurability, density, and diversity. Our previous investigations (Franěk, 2013; Franěk and Režný, 2014) examined the temporal aspects of the relationships between walking behavior and environmental properties. The idea is that an individual reacts to the surrounding environment in two ways—he/she is either trying to establish contact with the environment and stay inside it (approach behavior) or to avoid such contact and move away (avoidance behavior), which results in small fluctuations in walking speed. This idea is based on the approach-avoidance theory described by Mehrabian and Russell (1974).

In a previous series of experiments, we asked participants to walk along an urban route and observed that fluctuations in walking speed on the route were influenced by the environmental features of the urban areas the participants passed through (Franěk, 2013; Franěk and Režný, 2014). The route consisted of streets with various amounts of vegetation and various degrees of noise and traffic intensity. In general, the data revealed a tendency that participants tended to walk slightly faster in sections without greenery and with a higher level of traffic and noise than in sections with greenery and with a low level of traffic and noise. These tendencies became the starting point for the present research into the effect of music listening while walking in a real urban environment. In our previous experiments, we identified typical temporal walking patterns corresponding to particular sections of the route with certain environmental features. Thus, the next step was to provide the participants with music while walking along the route and observe whether listening to music could mask the impact of the surrounding environment and change the observed walking speed fluctuation patterns related to specific environmental properties.

The goal of the current study was to investigate the temporal aspects of walking with music under naturalistic conditions. The experiment addressed three questions:

1. The above-mentioned studies (e.g., Styns et al., 2007; Leman et al., 2013) described instructed synchronization of walking to music. It seems that people can synchronize to fast music in the course of various sport activities (e.g., Karageorghis et al., 2009). However, there is still the unanswered question of whether people synchronize spontaneously their step tempo to music from their portable music players while walking in a natural setting.
2. Both Karageorghis et al. (2009) and Leman et al. (2013) demonstrated the effect of *motivational* or *activating* music on bodily actions. This effect does not only depend on fast tempo but also on the sonic features of the music (Leman et al., 2013). We questioned whether this type of music can also motivate walkers in outdoor urban environments to walk faster or slower and suppress the effect of environmental properties.
3. Results in previous experiments (Franěk, 2013; Franěk and Režný, 2014) indicate that walkers without music respond to certain environmental features of the walking route by slightly speeding up or slowing down. The question is whether people walking with music show the same tendencies toward walking faster or slower depending on the visual properties of the environment. In other words, we questioned whether aural stimuli (music listening) could mask the effects of visual stimuli (i.e., the environmental features of particular sections of the urban walking route).

### Ethics statement

The research was in accordance with the Declaration of Helsinki. The participants provided verbal informed consent to participate. They declared that they were voluntarily participating in the experiment and that they were informed about the experimental task, the procedures, and the apparatus used. They agreed that video recordings of their actions and data from the questionnaires would be registered and used for scientific purposes only. They were allowed withdraw from the study at any moment. Their security was guaranteed, as the outdoor environment and tasks were not dangerous.

## Experiment 1

The present experiment was designed to study whether people walking with music show the same tendencies of walking faster or slower depending on the visual environment as when walking without music. At the same time, we were able to study whether subjects would spontaneously synchronize their walking to the music, as the participants thought that the study was about walking in a city and no particular attention was given to the music. The walking route was located in a downtown area of Hradec Králové, and it consisted of sections with various amounts of greenery and traffic.

In order to maximize the range of natural walking pacing mentioned in the literature (Styns et al., 2007), it is necessary to give the participants music with a tempo that does not deviate too much from the tempo of their normal walking tempo without music. When explicitly asked, individuals can synchronize with musical tempi within the range 50–190 bpm (Styns et al., 2007). However, we do not know in which range people would synchronize spontaneously. For practical reasons we processed three musical tracks consisting of identical musical pieces with 8% tempo difference (10% is the maximum amount that one can stretch or compress the tempo of existing music without a noticeable distortion of the signal, see Moens et al., 2010). The average tempo of the track was 114 bpm, 124 bmp, and 133 bpm, respectively.

Thus, in this experiment, prior to investigating the effect of music, we determined in no-music condition the basic effect of the environmental properties of the route on walking behavior and the mean walking tempo of the subjects. In the subsequent music condition, the participants received headphones and a mp3 player with a musical track in a particular tempo range. The tempo was assigned according to their average walking tempo from the no-music condition.

### Methods

#### Participants

In the no-music condition, 79 undergraduates attending psychology courses participated in the study. The students were young adults, 19–25 years old (*M* = 19.9 years, *SD* = 1.20), and the sample was composed of 47 men and 32 women. Nine undergraduates participating in the no-music condition failed to continue the experiment with the music condition. Thus, 72 undergraduates participated in the music condition (*M* = 20.2 years, *SD* = 1.22, 42 men and 30 women).

#### Walking route

The walking route was a 1.8 km circuit through various environments in the downtown area of the city of Hradec Králové in the Czech Republic. The route was located close to the university building. The participants knew the area. Because of the diverse characteristics of the specific environments within the route, the walking circuit was divided into 16 sections where we measured walking speed separately. The route and the sections are described in Table 1.

**Table 1:** **Walking route**.

**Section**	**Length (m)**	**Environmental layout**	**Street**
1	50	Traffic, no greenery	Gočárova
2	50	Traffic, no greenery	Gočárova
3	50	Weak traffic, trees	Nerudova
4	40	Weak traffic, old large trees	Mánesova
5	50	Busy traffic, trees, greenery	Střelecká
6	50	Weak traffic, no greenery	Nerudova
7	36	Weak traffic, greenery	Mánesova
8	35	Weak traffic, park	Mánesova
9	40	Weak traffic, greenery	Mánesova
10	75	Weak traffic, trees, greenery	Labská kotlina
11	50	Weak traffic, greenery	Labská kotlina
12	50	No traffic, park	Tylovo nábřeží
13	30	No traffic, park	Tylovo nábřeží
14	50	No traffic, park	Tylovo nábřeží
15	50	No traffic, park	Tylovo nábřeží
16	46	No traffic, park	Tylovo nábřeží

*The particular sections where the walking speed was measured*.

#### Procedure

The participants obtained a map of the route a week before the date of the study. The route was marked by noticeable arrows painted on the surface of a sidewalk to increase the ease of orientation. The participants walked the route individually. They were successively sent to start the route at intervals of approximately 5 min. They completed walking the route with the no-music condition first. On the following day (or in some cases, approximately 5–6 h later), they took part in the music condition. We estimated that the order of conditions (no-music condition first, music condition second) would not have a substantial influence on the results. First, the experimental task (walking) is common and cannot be improved by one experimental session. Second, the participants were familiar with the environment of the walking route.

The participants were instructed to walk along the route at their normal walking speed. In addition, they were asked not to stop during their walk and not to call or speak with other people. In the music condition, the participants were informed that they would receive a mp3 player with music. However, they were not explicitly instructed to synchronize with it. The music was played with an mp3 player Philips GoGear Raga 2 GB and Sony Dynamic Stereo Headphones MDR—V150. In the music condition, the participants have headphones, while in the no-music condition they walked without them.

Two directions of the walk along the route were employed in the experiment: direction A (from section 1 to section 16) and direction B (from section 16 to section 1). The study was conducted in 2010 over three working days: October 19th, October 20th, and October 21st. October 19th was cloudy, and the temperature was approximately 7°C. On the morning of October 20th, it was partly cloudy, and the temperature was approximately 4°C. There was a slight rain at approximately noon. During the afternoon, it was sunny, and the temperature was 7°C. On the morning of October 21st, it was windy and partly cloudy, with mild rainfall. The temperature was 2°C. In the afternoon, it was windy and partly cloudy, and the temperature was 5°C.

#### Musical tracks

Musical tracks A, B, and C with three different tempi were prepared. After completing the no-music condition, we immediately measured the average walking speed of a participant, and according to his/her walking speeds in the no-music condition, we assigned him/her a tempo of music close to his/her average walking speed (the method of measuring of the average walking speed is described in the subsection “Measurement of walking speed”).

Thirty-three participants (20 males and 13 females, the average bodily height was 177.7 cm, *SD* = 9.10) listened to music track A (Music A), with an average tempo of 114 beats/min (bpm). Thirty-five participants (20 males and 15 females, the average height was 177.2 cm, *SD* = 9.03) listened to music track B (Music B), with an average tempo of 124 bpm. Four participants (2 males and 2 females, the average height was 172.3 cm, *SD* = 14.98) listened to music track C (Music C), with an average tempo of 133 bpm.

World pop music with a clear beat and a stable tempo was selected. The music track consisted of nine pieces, all lasting approximately 3 min with no pause between them (see Table 2). In some cases, we took only the stable excerpts starting after the intro. The selected musical pieces were used in Music B at the original tempo; Music A was the same as Music B, although the tempo was 8% slower. Music C was the same as Music B, although the tempo was increased by 8%. The pitch was unchanged in Music A, B, and C. The participants listened to the music with a mp3 player.

**Table 2:** **Musical pieces used in the music condition of Experiment 1**.

**Experiment 1**			
**Track No.**	**Song**	**Artist/band, album, year**	**Tempo (bpm)**
1	Al Vaivén Mi Carretara	Nico Saquito, Good Bye Mr Cat, 1982	121
2	A deeper love	Aretha Flanklin, Greatest Hits, 1980–1994	121
3	Avalon	Jacques Lu Cont vs. Remix, 2005	127
4	Sahib Balkan	Buscemy, Gypsy beat, and Balkan bangers, 2006	128
5	Crystal frontier	Calexico, Bucemi remix, 2006	121
6	Shake it	Extended Mix, Lee Cabrera, Housworks Boom The Ultimative Hits, 2010	128
7	Vino vino	Ian Oliver and Eastenders, Vino Vino, 2008	125
8	Kiss my eyes	Bob Sinclair, US Hot Dance Music/Club Play, 2003	126
9	Istanbul Çocuklari	Baaba Zula and Mad Professor, Duble Oryantal, 2005	121

*The selected musical pieces were used in Music B at the original tempo. Music A was 8% slower, music C was 8% faster*.

#### Measurement of walking speed

**Figure 1:**
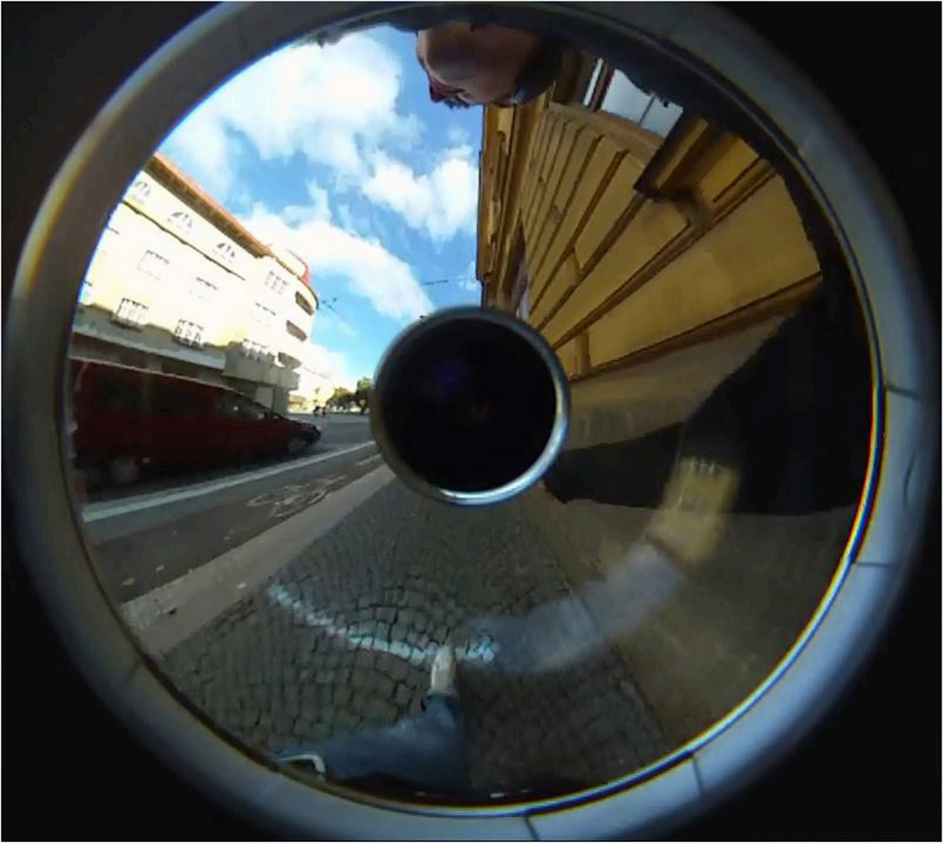
**Example of the video frame capturing a subject entering the track section**.

The subjects walked with a small video camera (Sony Bloggie MHS-PM5K) on a belt around their waist (size 19 × 108 × 55 mm, weight 110 g). Through a fish eye lens, the environment, the subject's feet, and the subject's arms were captured. The evaluation procedure consisted of annotating the video records in the software *Elan* (see https://tla.mpi.nl/tools/tla-tools/elan/). Each section of the route had its beginning and end clearly indicated by a line drawn with intense color on the sidewalk. An evaluator marked two frames of the video record to create begin and end of the annotation for each particular track section. Each frame corresponded to a time when a participant entered or left the section (see Figure 1). Whole procedure was performed in the software Elan during video playback and annotations comprised the name of the track section and were time-aligned to the video records. Annotations marked in the video record were later exported from the Elan. Every single annotation represented whole section of the track, determining how much time subjects spend there. This allowed an easy transfer of data directly to the spreadsheet software, where the results were supplemented with section lengths. This enabled calculation of the average speeds reached by the subjects in all sections. The video records were processed by a team of eight research assistants previously acquainted with the evaluating procedure.

#### Analysis of synchronization

The interstep times were analyzed from the video by first making a motion timeline by taking a line of pixels out of each video frame showing the region where the feet passes during walking. The resulting image was next analyzed by a Matlab program registering the regular changing of colors along the X-axis, reflecting the presence or absence of a foot. This procedure delivers the period of the stride. This period was compared with the period of the beat of the music. No exact phase alignment was obtained, but the stationarity of the walking period made it clear when the subject was synchronizing. In this case also a clear transition of the tempo was visible at the change of the musical pieces, which also changed the tempo from one piece to the other.

### Results and discussion

#### No-music condition

**Figure 2:**
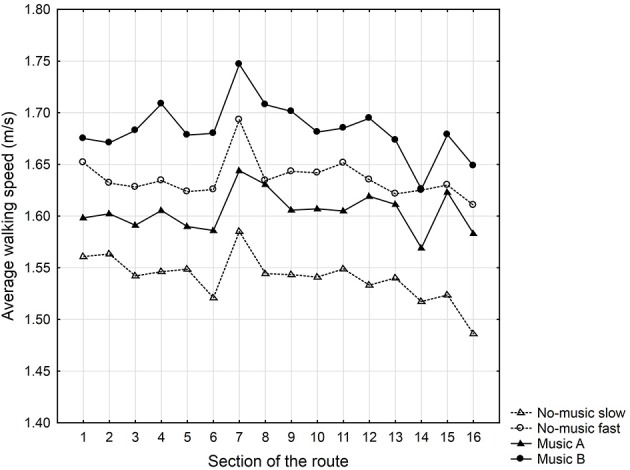
**Average walking speeds (m/s) in particular sections of the route for Experiment 1 (no-music condition and music condition with Music A and Music B)**. Participants indicated as “No-music slow” received Music A in the music condition, and participants indicated as “No-music fast” received Music B in the music condition.

Average walking speeds in particular sections of the route were calculated (see Table 3 and Figure 2). A repeated measures analysis of variance (ANOVA) was carried out to determine the effects of particular variables on the walking speed in the particular sections. Participants' gender and direction of the walk on the route were chosen as independent variables. The section of the route was a repeated measure factor, and the speed of walking was the dependent variable. The ANOVA indicated a statistically significant within-subjects main effect of the section walked [*F*_(15, 1035)_ = 7.18, *p* < 0.001, η^2^ = 0.09] and a statistically significant between-subjects main effect of gender [*F*_(1, 1035)_ = 13.78, *p* < 0.001, η^2^ = 0.17]. There were also statistically significant interactions between the section and the direction of the walk [*F*_(15, 1035)_ = 3.23, *p* ≤ 0.001, η^2^ = 0.04] and among section, gender, and the direction of the walk [*F*_(15, 1035)_ = 1.95, *p* ≤ 0.05, η^2^ = 0.03]. The Tukey Honestly Significant Difference (HSD) *post-hoc* test revealed significant differences in walking speed between section 7 and all other sections (Cohen's *d* ranged from 0.22 to 0.57) as well as between section 16 and all other sections (Cohen's *d* ranged from 0.07 to 0.57). Thus, the effect sizes ranged from small to medium.

**Table 3:** **Average walking speeds (m/s) in particular sections of the route for Experiment 1 (no-music condition and music condition with Music A and Music B) and Experiment 2 (two music conditions: motivational music and non-motivational music)**.

**Section**	**Experiment 1**						**Experiment 2**			
	**Non-music condition**		**Music condition**							
			**Music A**		**Music B**		**Motivational m.**		**Non-motivational m.**	
	***M***	***SD***	***M***	***SD***	***M***	***SD***	***M***	***SD***	***M***	***SD***
Section1	1.61	0.13	1.59	0.13	1.68	0.12	1.66	0.15	1.51	0.17
Section 2	1.60	0.13	1.60	0.11	1.67	0.13	1.67	0.17	1.49	0.17
Section 3	1.59	0.13	1.59	0.11	1.68	0.14	1.68	0.18	1.46	0.17
Section 4	1.59	0.14	1.61	0.12	1.71	0.16	1.66	0.18	1.45	0.16
Section 5	1.59	0.13	1.59	0.12	1.68	0.12	1.66	0.16	1.45	0.16
Section 6	1.57	0.14	1.59	0.13	1.68	0.13	1.66	0.18	1.43	0.18
Section 7	1.64	0.14	1.64	0.11	1.75	0.14	1.70	0.27	1.46	0.17
Section 8	1.59	0.13	1.63	0.13	1.71	0.14	1.71	0.26	1.48	0.16
Section 9	1.59	0.13	1.61	0.13	1.70	0.14	1.68	0.22	1.46	0.16
Section 10	1.59	0.14	1.61	0.12	1.68	0.13	1.67	0.16	1.47	0.17
Section 11	1.60	0.13	1.60	0.14	1.69	0.12	1.66	0.17	1.46	0.17
Section 12	1.59	0.14	1.62	0.13	1.69	0.12	1.67	0.18	1.47	0.17
Section 13	1.59	0.14	1.61	0.14	1.67	0.11	1.67	0.15	1.48	0.17
Section 14	1.57	0.14	1.57	0.17	1.63	0.14	1.66	0.15	1.46	0.18
Section 15	1.58	0.15	1.62	0.13	1.68	0.12	1.67	0.16	1.49	0.16
Section 16	1.56	0.14	1.58	0.13	1.65	0.13	1.68	0.14	1.50	0.15
Average speed on the route	1.59	0.14	1.60	0.13	1.68	0.13	1.67	0.17	1.47	0.14

The results indicated that average walking speeds in the specific sections differed. Participants walked faster on streets without greenery and higher amounts of traffic (sections 1 and 2), streets with a high amount of traffic but a high amount of greenery (section 5) and streets with a low amount of traffic but a lack of greenery (section 11). In contrast, a slower walking speed was observed in the sections with a park design (sections 13, 14, and 16). Surprisingly, a suspicious increase in walking speed appeared in section 7 in both directions. It is possible that the acceleration was a response to the unkempt environment in this section (i.e., a dirty facade on one side of the street and a damaged facade on the opposite side) in contrast with the clean and well-maintained facades in other sections of the route. However, a deeper analysis and discussion of the potential impact of particular environmental properties on behavioral reactions is beyond the scope of this paper. The patterns of walking speeds within the route agree with findings from our previous investigations (Franěk, 2013).

Females, on average, walked slower (mean = 1.54 m/s, *SD* = 0.02) than males (mean = 1.62 m/s, *SD* = 0.02). It is evident that the velocities reflect different body height. The average height of males was 180.9 cm (*SD* = 7.35), while average height of females was 168.3 cm (*SD* = 6.17). Although there were no differences between patterns of walking speed in direction A (from section 1 to section 16) and direction B (from section 16 to section 1) in males, females walked faster in direction A than in direction B. Gender differences in walking behavior explain both the significant interaction between the section and the direction of the walk, as well as significant interaction among section, gender, and the direction of the walk. We assumed that this difference might be caused by the effect of temporal priming—in direction A (the route begins with busy streets without greenery), the participants started to walk faster, and this initial walking speed may have influenced the pace of the whole route. In contrast, females who walked in direction B (the route starts in a small park) started to walk relatively slowly, which resulted in an overall slower walking speed within the route. It seems that females were more sensitive to the environment than males.

#### Music condition

First, we analyzed synchronization between the step tempo and the tempo of the musical pieces (see Table 4). The tracks of 9 pieces were available to the participants on their mp3 players; however, most participants finished their walk in the 6th or 7th song on the list. Thus, songs 8 and 9 on the list were never used. The results showed (see Table 4) that during the first song, there was not much synchronization. This may be because it was the first song, or it could be because the music was more difficult to synchronize to. Song 7 was the last song, and most people were finished with their walk before this song played. Thirty-eight subjects did not synchronize with any song. Very few subjects synchronized with song 3, 4, or 6. The synchronization was most frequent with song 5. A detailed analysis of synchronization with music for each participant is available in the *Supplementary Material*. To sum up, the results showed that many subject did not spontaneously synchronize with the beat of the music at all, and some subjects synchronized only part of the time.

**Table 4:** **Synchronization between the step tempo and the tempo of musical pieces in Experiment 1**.

	***N***	**Synchronized with song number:**							**Sum of synchronized songs**							
		**1**	**2**	**3**	**4**	**5**	**6**	**7**	**0**	**1**	**2**	**3**	**4**	**5**	**6**	**7**
Walking slower with music	5	2.0	2.5	1.0	2.0	2.5	1.5	1.0	3			1			1	
Partly slower, partly equal	3	0.0	2.0	0.0	0.5	1.0	0.0	0.0	1	1	1					
Partly slower, partly faster	11	1.0	3.0	5.5	4.0	5.5	6.0	1.0	2	2	2	4		1		
No influence of music on walking tempo	18	0.5	0.5	0.5	2.0	2.5	0.5	1.0	17							
Walking faster with music	22	2.5	8.5	7.0	4.0	7.0	5.5	0.0	10	4	4	3	1	1		
Partly faster, partly equal	11	0.0	3.5	1.0	5.0	4.0	1.5	0.0	5	4	1	1				
Total	70	6.0	20.0	15.0	17.5	22.5	15.0	3.0	38	11	8	9	1	2	1	0

*The left section provides a general description. The middle section indicates the songs for which there was synchronization (songs 1–7). Synchronization was scored at two levels: (1) clearly synchronizing and (2) possibly synchronizing. A full point was given to the clear synchronizers, and a half a point was given to the possible synchronizers. The right section shows the number of songs for which synchronization was observed*.

In the next analysis, we examine the effect of music tempo on the walking speed. The average walking speeds in particular sections are shown in Table 3 and Figure 2. Because only four participants walked with Music C, we omitted this group from the statistical analyses. A repeated measures analysis of variance was carried out to indicate the effects of particular variables on the walking speed in the particular sections. Participants' gender, direction of the walk on the route, and music track (Music A vs. Music B) were chosen as independent variables. The section of the route served as a repeated measure factor, and walking speed was the dependent variable. The ANOVA indicated a statistically significant within-subjects main effect of section walked [*F*_(15, 750)_ = 4.976, *p* < 0.001, η^2^ = 0.09] and a statistically significant between-subjects main effect of music track [*F*_(1, 750)_ = 3.943, *p* = 0.05, η^2^ = 0.07]. Gender and direction of the walk on the route had no significant effects.

Participants listening to Music A showed an average walking speed throughout the entire route of 1.60 m/s (*SD* = 0.13). The average walking speed of participants listening to Music B was 1.68 m/s (*SD* = 0.13). Cohen's *d*, which describes the effect size of both musical tracks, was 0.62. The results showed that the tempo of the music track had some effect on the overall walking speed. Participants who listened to faster music while walking (Music B) walked faster than those listening to music with a slower tempo (Music A). However, Music A was assigned to individuals who were “slow walkers” in the no-music condition, and Music B was assigned to individuals who were “fast walkers” in the no-music condition; therefore, this difference may not to be due to the influence of music itself. To properly determine the effect of music listening while walking, we compared the walking speeds of “slow walkers” and “fast walkers” in no-music and music conditions (see Figure 2). We found that both “slow walkers” and “fast walkers” walked faster in the music condition than in the no-music condition. There was a significant difference between no-music and music conditions for the “slow walkers” [*F*_(1, 63)_ = 5.104, *p* < 0.05, Cohen's *d* = 0.462], while only a non-significant difference was observed for the “fast walkers” [*F*_(1, 69)_ = 2.922, *p* = 0.09, Cohen's *d* = 0.333). This indicates that music listening while walking was motivating, especially for “slow walkers,” as they took longer steps in the condition with music compared the no-music condition. We suppose the smaller and the non-significant effect of music in “fast walkers” was caused by physical constraints of walking speed: participants reached the comfort level of their normal walking speed. To make even bigger steps and walk faster could be uncomfortable, while running could be inappropriate in the urban context.

Finally, we examined whether listening to music with headphones can mask the effects of properties of the visual environment. For the participants walking with Music A, the Tukey HSD *post-hoc* test revealed significant differences in walking speed between sections 7 and 14 (Cohen's *d* was 0.34). For the participants walking with Music B, the Tukey HSD *post-hoc* test yielded significant differences in walking speed between section 7 and the other sections (except sections 4, 8, and 9; Cohen's *d* ranged from 0.46 to 0.86) and significant differences between section 16 and sections 4, 7, and 8 (Cohen's *d* ranged from 0.15 to 0.74). The effect sizes ranged from small to large. Thus, similar to what was observed in the no-music condition, we observed that visual environmental properties had a significant effect on walking speed in several sections of the route in the music condition. Moreover, the data showed similar patterns of walking speed fluctuation within the route in both the music and no-music conditions. Specifically, the increase in speed in section 7 was very suspicious. It seems that listening to music with headphones while walking did not mask the effect of environmental stimuli, which was registered in the previous experiment. A possible explanation is that participants had to pay a great deal of attention to the proper route because they were walking in a city environment with a certain level of traffic and movement of other pedestrians.

However, the present experiment did not clearly proved, whether the walking behavior was influenced only by music masking or by acoustical masking given by the fact that participants had the headphones. The subsequent experiment conducted in the same conditions as the music condition of the present experiment would better answer to the phenomenon of masking of the environmental properties related to the music listened while walking.

## Experiment 2

In the present experiment we intended to increase the effect of music on walking with explicitly determined motivational and oudeterous music (see Karageorghis et al., 1999). We questioned whether the influence of the visual properties of an environment would still present in these extreme cases. Because motivational music should induce bodily action, we expected that this music would have a greater effect on walking behavior. To serve as a contrast to motivational music, we also used non-motivational music. While Karageorghis' concept of oudeterous music states that this music should be neutral in terms of motivational qualities, the second type of music used in this study, non-motivational music, was characterized as nice music; however, it did not stimulate the subject to move. The participants were asked to listen to either motivational or non-motivational music while walking the route identical to the route employed in Experiment 1.

This experiment explored how the tempo of either motivational or non-motivational music while walking influenced the tempo of the participants' walking speed. The next question was, whether listening to either motivational or non-motivational music could better mask the effects of environmental visual stimuli observed in Experiment 1. The last question was whether any individual differences exist in individuals' reactions to the music listened to while walking. Hence, the scores of five personality traits were measured.

### Methods

#### Participants

A total of 121 undergraduates attending psychology courses participated in the study. The students were young adults, 19–32 years old (*M* = 20.6 years, *SD* = 1.57), and the sample was composed of 61 men and 60 women.

#### Musical stimuli

Approximately 1 month before the experiment, the participants were asked to select and submit two files of different types of music that they liked. The first type of music, motivational music, was characterized as “Music that gives me a strong urge to move in one way or the other,” while the second type of music, non-motivational music, was described as “Nice music, but with no strong urge to move.” The musical files had durations of approximately 3 min.

Further, approximately 2 weeks before the experiment, the participants were asked to evaluate the motivational character of these collected musical files, which were made available on a network disk, using the Czech version of the Brunel Music Rating Inventory-2 (Karageorghis et al., 2006). The inventory consisted of five items: (1) The rhythm of this music would motivate me during exercise. (2) The style of this music (i.e., rock, dance, jazz, hip-hop, etc.) would motivate me during exercise. (3) The melody (tune) of this music would motivate me during exercise. (4) The tempo (speed) of this music would motivate during exercise. (5) The sound of the instruments used would motivate me during exercise. Participants were required to rate the level of their agreement/disagreement with these items using seven-point Likert-type scales ranging from 1 (Absolutely disagree) to 7 (Absolutely agree). The files of “motivational music” and “non-motivational music” were evaluated separately.

Based on evaluation using the Brunel Music Rating Inventory-2, we selected nine musical pieces with the highest motivational character (motivational music) and an additional nine pieces with the lowest motivational character (non-motivational music). Then, we constructed two musical tracks that were used in the experiment. The first musical track, Music 1 (motivational music), consisted of energetic dance music, with clear and strong percussive, often electronic accompaniment. The second musical track, Music 2 (non-motivational music), consisted of ballads and “sing-a-song” style mood music (see Table 5). The music was presented to the subjects with a mp3 player.

**Table 5:** **Musical pieces used in Experiment 2**.

**Track No.**	**Song**	**Artist/band, album, year**	**Tempo (bpm)**	
**MOTIVATIONAL MUSIC**				
1	Danza Kuduro	Don Omar, Lucenzo, Meet the Orphans, 2010	131	
2	American idiot	Green Day, American Idiot, 2004	151	
3	Mambo no. 5	Lou Bega, A Little Bit of Mambo, 1999	135	
4	Mr. Saxobeat	Alexandra Stan, Saxobeats, 2011	158	
5	One fine day	The Offspring, Conspiracy of One, 2000	187	
6	Crash	Matt Willis, movie “Mr. Beans Holiday,” 2007	192	
7	Greased lightning	John Travolta, movie “Grease,” 1978	200	
8	Don't stop me now	Queen, Jazz, 1979	200	
9	On the floor	Jennifer Lopez, Pitbull, Love?, 2011	140	
**NON-MOTIVATIONAL MUSIC**				
1	Deadmen's gun	Ashtar Command, video game “Red Dead Redemption,” 2010	69	
2	May it be	Enya, movie “The Lord of the Rings, the Fellowship of the Ring,” 2001	52	
3	Fix you	Coldplay, X and Y, 2005	63–84	The tempo was not constant
4	The time machine	Klaus Badelt, movie “The Time Machine,” 2002	96	
5	Mad world	Michael Andrews, movie “Donnie Darko,” 2001	91	
6	C'est la mort	Krucipüsk, Druide!, 2004	75–78	The tempo was not constant
7	Now we are free	Lisa Gerrard, movie “Gladiator,” 1981	74–82	The tempo was not constant
8	Only time	Enya, A Day Without Rain, 2001	52–53	The tempo was not constant
9	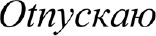	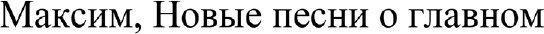 , 2007	60–65	The tempo was not constant

#### Personality measurement

Personality was measured using Costa's and McCrae's NEO Five-Factor Inventory (the Czech translation by Hřebíčková and Urbánek, 2001). The inventory consists of 60 items and measures five personality traits: neuroticism, extraversion, openness, agreeableness, and consciousness.

#### Procedure

The participants walked the route, which was identical to the route in Experiment 1, individually. They were successively asked to start the route at intervals of approximately 5 min. Two directions of walking along the route were employed: Direction A (from section 1 to section 16) and Direction B (from section 16 to section 1). The participants were instructed to walk at their normal walking speed. Moreover, they were informed that music would be presented to them with a mp3 player; however, they were not instructed on what to do with it. Approximately 1 week after completing Experiment 2, the participants were asked to fill out Costa's and McCrae's NEO Five-Factor Inventory.

A between-subjects design was used. The participants were assigned either the motivational music or the non-motivational music. All conditions were balanced between males and females. Thirty males and thirty-two females were assigned the motivational music, while thirty-one males and twenty-eight females were assigned the non-motivational music.

The study was conducted in 2011 over the span of three working days: November 8th, November 9th, and November 10th. November 8th was cloudy, with a temperature between 10 and 11°C. On the morning of November 9th, it was cloudy, and the temperature was between 8 and 11°C. In the afternoon, it was sunny, and the temperature was 12°C. On the morning of November 10th, it was cloudy, with a temperature of 6°C. In the afternoon, it was partly cloudy, and the temperature was 6°C.

The method of walking speed measurement was the same as that described in Experiment 1.

### Results and discussion

Synchronization between the tempo of the music and the step tempo was not analyzed. Music 1 was too fast for normal walking. Only one subject that started to run was synchronizing. In contrast, Music 2 was too slow for normal walking.

**Figure 3:**
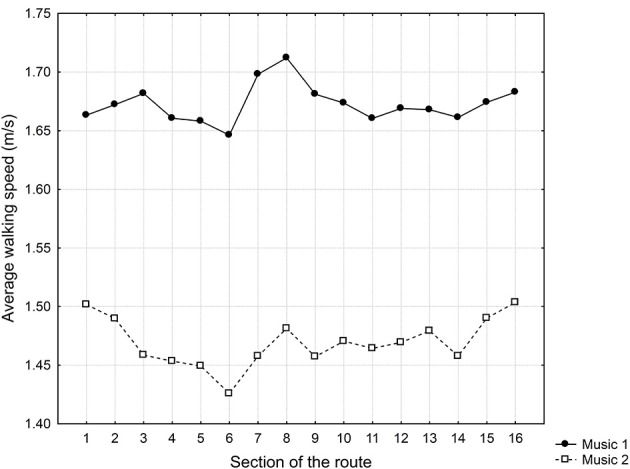
**Average walking speeds in particular sections of the route for Experiment 2**. The results for two music conditions are plotted: Music 1 (motivational music) and Music 2 (non-motivational music).

The average walking speeds in the particular sections are shown in Table 3 and Figure 3. Repeated measures ANOVA was carried out to indicate the effects of particular variables on the walking speed in particular sections. Participants' gender, direction of the walk on the route, and type of music were chosen as independent variables. The section of the route served as a repeated measure factor, and walking speed was the dependent variable. The ANOVA indicated a statistically significant between-subjects main effect of the type of music [*F*_(15, 1485)_ = 43.72, *p* < 0.001, η^2^ = 0.31] and a statistically significant within-subjects main effect of the section walked [*F*_(15, 1485)_ = 4.60, *p* < 0.001, η^2^ = 0.04]. There were significant interactions among section, direction of walk, and gender [*F*_(15, 1485)_ = 1.69, *p* < 0.05, η^2^ = 0.02] and among type of music, section, direction of walk and gender [*F*_(15, 1485)_ = 1.92, *p* < 0.05, η^2^ = 0.02].

In the next analysis, we examined the effect of the tempo of music on the walking speed. The results indicated that the type of music (motivational vs. non-motivational) had a strong effect on walking speed in particular sections. The average walking speed of participants listening to Music 1 (motivational music) throughout the entire route was 1.67 m/s (*SD* = 0.17), while the average walking speed of participants listening to Music 2 (non-motivational music) was 1.47 m/s (*SD* = 0.14). The effect size was high (Cohen's *d* = 1.290). On average, females walked slower than males with both types of music. This difference was particularly notable in the direction of walk A.

The environmental properties of the different sections had a substantially smaller impact on walking speed. The Tukey HSD *post-hoc* test revealed significant differences in walking speed between sections 6 and all other sections except for sections 4 and 5 (Cohen's *d* ranged from 0.06 to 0.22 for motivational music and from 0.17 to 0.46 for non-motivational music) as well as between section 8 and sections 4, 5, 6, 11, and 14 (Cohen's *d* was 0.22 for motivational music, and for non-motivational music, it ranged from 0.12 to 0.29). Thus, the effect sizes were mostly small. The pattern of fluctuation in walking speeds in particular sections of the route was different than that observed in Experiments 1. In contrast with the data from Experiment 1, the effects of the green environment completely disappeared. There was a suspicious increase in speed in section 8, which may be a reaction to unexpected repair work on the facade of a house on the opposite site of the street. Most likely, this work activity on the calm street led the participants to speed up. In contrast, the suspicious slowing down in section 6 may be due to the narrowing of the street in one place (in contrast to Experiment 1, in which the participants were walking on the opposite side of the street in this section due to technical reasons). Non-significant slowing in this section was also observed in Experiment 1. It seems that both types of music required more attentional resources, and slower walking speeds were required to pass through this constricted section. However, in general, both types of music masked the influence of the visual characteristics of the environment to a greater extent than what was observed in Experiment 1.

Although motivational and non-motivational music differed in their tempi, we assume that both types of music affected the participants during their walk more than music in Experiment 1. The motivational music affected the participants by its energetic character and fast tempo, while in contrast, the non-motivational music by its relaxing and serene character. The stronger effect of the music on walking behavior was also noticed during our observations of the participants walking on the route (see below). Moreover, the musical tracks consisted of pieces which were selected by our participants. Thereby one can suppose that the music used in Experiment 2 was closer to musical preferences of the participants. It is worth commenting that our participants represented a relatively culturally homogenous group. Almost all of them came from the same region, their families belonged to lower-middle class, they were mostly not active musicians, and their study interests were oriented to informatics and business.

Finally, we were interested in associations between some personality traits and walking behavior. The effects of neuroticism and extraversion were analyzed. The average neuroticism score for males was 19.79 (*SD* = 7.81); for females, this score was 23.88 (*SD* = 6.77). The average extraversion score for males was 31.39 (*SD* = 7.83); for females, this score was 33.43 (*SD* = 5.47). Compared to the age-matched general population in the Czech Republic, our participants were slightly less neurotic and slightly more extraverted (see Hřebíčková and Urbánek, 2001). Correlations among the average speed throughout the whole route, the speeds in particular sections of the route, and scores of neuroticism and extraversion were computed (see Table 6). Although the correlation coefficients were not high and some of them were not significant, they showed that personality was associated with individuals' reactions to motivational and non-motivational music. In walking with motivational music, we found negative correlations between walking speed and neuroticism; however, only two of these correlations were significant. It indicates that there was a slight tendency in participants with a higher level of neuroticism to walk slowly while listening to motivational music. The correlations between walking speed and extraversion were below zero and non-significant. In walking with non-motivational music, the correlations between neuroticism and walking speed were positive but non-significant. Interestingly, the effect of neuroticism was opposite compared to walking with motivational music, despite the lack of significance in the results. There were negative correlations between extraversion and walking speed, and many of them were significant. This result indicates that there was a tendency in participants with higher levels of extraversion to walk slower while they were listening to non-motivational music. The correlations between the walking speed and scores of the other personality traits of the Big Five model (openness, agreeableness, and consciousness) were about zero and were not significant.

**Table 6:** **Correlations between walking speeds in particular sections of the route and scores of neuroticism and extraversion in Experiment 2**.

**Walking speed in particular sections**	**Motivational music**		**Non-motivational music**	
	**Neuroticism**	**Extraversion**	**Neuroticism**	**Extraversion**
Section 1	−0.08	−0.06	0.24	−0.26
Section 2	−0.12	−0.03	0.18	−0.23
Section 3	−0.22	0.04	0.22	−0.26
Section 4	−0.23	0.01	0.19	−0.23
Section 5	−0.19	−0.06	0.17	−0.22
Section 6	−0.17	−0.06	0.18	−0.21
Section 7	−0.12	0.02	0.21	−0.24
Section 8	−0.14	0.01	0.25	−0.29^*^
Section 9	−0.18	0.02	0.21	−0.23
Section 10	−0.27	−0.08	0.22	−0.27
Section 11	−0.27^*^	0.00	0.17	−0.24
Section 12	−0.27^*^	−0.05	0.17	−0.30^*^
Section 13	−0.21	−0.04	0.21	−0.35^*^
Section 14	−0.17	−0.08	0.20	−0.36^*^
Section 15	−0.20	−0.06	0.17	−0.36^*^
Section 16	−0.20	−0.07	0.19	−0.32^*^
Average speed on the route	−0.20	−0.03	0.21	−0.29^*^

*Boldfaces indicate significant correlations*,

* *p < 0.05*.

In general, motivational music led participants to walk faster in agreement with the dynamic character of the music, while non-motivational music led participants to walk slower, in a relaxed way. However, our data revealed also certain individual differences. It appeared that participants with higher levels of neuroticism did not react to the dynamic character of motivational music in a similar way as participants with a lower level of neuroticism. It is possible that participants with higher levels of neuroticism were hesitant to express their behavioral responses to music openly in a public space; thus, they tended to suppress the effect of motivational music. Contrariwise, the participants with a higher level of neuroticism walked faster than those, who scored low in this personality trait (although the association was non-significant) while listening to non-motivational music. We assumed that this reaction reflected the reluctance to express behavioral responses to slow and moody non-motivational music and to walk slower, in a relaxed way.

There was no association between a level of extraversion/introversion and walking speed in the motivational music condition. Despite the correlation between walking speed and the score of extraversion was negative, it was very close to zero and non-significant. However, extraversion was significantly associated with slower walking while listening to non-motivational music. It means that a higher level of extraversion made easier to react appropriately to slow and moody character of the non-motivational music and walk on the streets suspiciously slower, in a relaxed way.

Although we did not conduct a systematic observation of our participants during their walk on the route, random observations of some walkers showed that motivational music substantially changed walking behavior. In contrast with ordinary pedestrians, the participants listening to motivational music walked considerably faster and more energetically, while those listening to non-motivational music were considerably slower and relaxed compared to other pedestrians. For instance, we found that participant P. P., who was listening to motivational music, was running in three sections of the route (the song *Mr. Saxobeat* was played, see Table 5). When asked about the experience, she explained that motivational music was very nice and it made her very happy; thus, she had a need for faster movement. Then, we checked her extraversion score, which was quite high (*E* = 35). In contrast, participant Z. J., who listened to non-motivational music, was found walking very slowly on the track and, notably, also swinging a bit. The song *Only time* was played (see Table 5). His level of Extraversion was also high (*E* = 35), and his level of neuroticism was very low (*N* = 12).

## General discussion

This study is the first to explore spontaneous temporal aspects of walking with music in naturalistic conditions. In studies on beat-synchronized walking, synchronization was often reached by instructing participants to synchronize with the music (Styns et al., 2007; Leman et al., 2013). However, our findings suggests that people in naturalistic conditions do not spontaneously synchronize with the beat tempo of the music. Although we chose music with a clear beat, which was selected according to participants' spontaneous walking tempo, synchronization occurred only occasionally. It seems that on an ordinary walk along urban streets, people are not comfortable changing their movement tempo and synchronize with the beat of the music. On the other hand, music does influence walking speed.

The results suggest that the energetic music has the activating effect and the non-energetic calm music has the relaxing effect. In Experiment 1 only the activating music was used, therefore, in general, the participants tended to walk faster than in the no-music condition. In contrast, in Experiment 2 the fast, energetic motivational music seems to induce people walk faster, while the non-motivational, slower, relaxing music makes people walk slower. This suggest that music influences gait tempo and step size but does not necessarily lead to precise synchronization.

It is worth commenting that the ability to synchronize walking tempo with music can be influenced by musical expertise of the participants. Our participants were not in most cases active musicians or musical experts. The music used in Experiment 1 was common popular music with a clear beat, thus we do not think that inducing the beat from the musical pieces would be difficult for non-musicians. In addition, the studies by Styns et al. (2007) and Leman et al. (2013) did not report any differences in the instructed synchronization of walking to music between musicians and non-musicians. Moreover, both Styns et al. (2007) and Mendonça et al. (2014) have also used pure rhythmical stimuli for a control (metronome stimuli or footsteps sounds), which did not require the participants to induce the beat from complex acoustical structures. They did not find any differences in synchronization between rhythmical stimuli and musical pieces. Styns et al. (2007) in their walking study also described the behavior of two participants who were not able to synchronize with faster musical pieces, but increased their walking tempo. The authors suggested that these participants experienced the musical stimuli as background music rather than the rhythmical stimulation for synchronization. It seems that this explanation could also be used to explain the behavior of our participants. We assume that participants in our experiments, who were not implicitly asked to synchronize their walking tempo with music, experienced the presented musical tracks as background music and did not consider it necessary to synchronize with them. They only synchronized occasionally, perhaps in situations when they liked the music, or the tempo of the music was convenient for them, or if they were not disturbed by external environmental stimuli. Because we did not have any control group of experienced and active musicians, we can only assume that these findings have general validity for people with various levels of musical expertise. Clearly, further research is needed.

The lack of synchronization with the music can be also explained by a feature of the outdoor environment. While synchronization studies were conducted in artificial environments (e.g., athletic halls, treadmill), in a real environment walkers have not an empty straight track about several 100 m long, where they can walk without any interruption. In the outdoor urban environment there are many disturbing stimuli, for instance other walkers on a sidewalk or cars driving down the street. The walkers also need to cross a crossroad, turn to another street, etc. All these stimuli or activities distract attention away from the music. This was also confirmed in laboratory conditions in the study by Sejdić et al. (2012), who investigated stride interval stationarity while watching TV. They reported that TV diminished their focus on walking, which resulted in non-stationary walking. There is another, still unsolved question, about whether an individual's walking speed may be influenced by the speed of other walkers or even the speed of cars driving down the street.

If we consider the factors affecting walking with music, we can also speculate about seasonal factors influencing mood. Curiously, the study by Pettijohn et al. (2010) reported seasonal music preferences in college students. They preferred reflexive and complex music when primed with fall/winter and energetic and rhythmic and upbeat and conventional music when primed with spring/summer. Both our studies were conducted in the fall, at the end of October or beginning of November. Would participants react in a different way on the activating music, which belongs to the categories “energetic and rhythmic” and “upbeat and conventional” music (Rentfrow and Gosling, 2003), in the spring?

Interestingly, in the most recent, yet not published study by Moens et al. (in press), which was partly conducted in naturalistic conditions on the same walking route as we used in the present experiments, the authors reported that participants were more successful in spontaneous synchronization between the music tempo and tempo of walking when the music's tempo and the user's pace were close enough to each other. D-jogger (Moens et al., 2010), the musical interface which was used in the experiment, matches the tempo of the music with the walking tempo of the user, switching songs when appropriate. It shows a great potential in future practical applications of the research in temporal aspects of walking behavior. As we already mentioned, a mobile system, which will enable flexible rhythmic stimulation, could be used not only for relaxation purposes (walking, jogging), but also for recovering features of movement performance.

As previously mentioned, it is supposed that listening to music while traveling or walking is often used to create an “auditory bubble,” which helps people to isolate themselves from unpleasant stimuli from the surrounding environment (Bull, 2001). However, we had no empirical data showing how effectively such an “auditory bubble” works and to what extent it can suppress surrounding environmental stimuli and information. Therefore, our study attempted to identify the extent of the effect of such an “auditory bubble” created by selected music. While it may be quite difficult to measure the level of perception of surrounding environmental stimuli, we utilized our findings related to the behavioral reactions of walkers to specific visual properties of the environment of a walking route, namely the pattern of walking speeds in the different sections. Therefore, we explored whether people walking with music listened through headphones show the same tendencies to vary their walking speed depending on the visual properties of the environment as people walking without music. We found that music did not have much effect. Music can, to some extent, mask the influence of the surrounding environment while walking. Of course, the effect of an “auditory bubble” is also influenced by the type of music, as documented by our data. Heye and Lamont (2010) performed a study of the effects of music and demonstrated how mobile listeners readily switched between inside (music) and outside (surrounding) worlds. The outside world tends to take priority when a conflict arises in a traveling situation. The distracting effect of visual information, which diverts the participant's attention from the walking task, was documented in several laboratory studies (Prokop et al., 1997; Sejdić et al., 2012). This can explain why listening to music through headphones in our experiments did not mask the influence of some notable environmental variables occurring along the walking route. Most likely the “auditory bubble” can suppress environmental stimuli more effectively when we are sitting in a train and listening to music. On the other hand, people who use the “auditory bubble” while traveling by public transport more likely do so to mask unwanted or unpleasant auditory stimuli, rather than visual ones, from the surrounding environment. There is the question of what was really masked in our experiments? Both our experiments, as well as the study by Heye and Lamont (2010), investigated walking with music in relatively silent areas. The streets with a high traffic in the down-town area of Hradec Králové are not as noisy as busy urban highways, for example. It seems that the effect of listening to music while walking was not to mask unwanted acoustical stimuli, but, more likely, to transform the surrounding environment into a private and pleasurable space. The recent study by Yamasaki et al. (2013) demonstrated how listening to music can change perception and evaluation of an environment. For instance, highly active music can increase the activation ratings of environments. Also, highly positive music can increase the positivity ratings of the environments.

It is worth commenting that it is also useful to look for individual differences in reactions to music while walking. Musical preference, as well as musical activity, could be important factors. It was also shown that some personality traits, namely extraversion and neuroticism, played some role. While neurotic and introverted individuals were more likely to use music for emotional regulation (Chamorro-Premuzic and Furnham, 2007), our data shows that the expression of emotions elicited by music and accompanied by music-related movements is easier for less neurotic people. However, in our data associations with extraversion were not entirely clear. Moreover, contrasting with the results reported by Chamorro-Premuzic and Furnham (2007), Chamorro-Premuzic et al. (2009) in the consequent study found that extraversion positively predicted using music for emotional regulation. Clearly, further investigations of music and personality are needed.

Although the present experiments, which were conducted in a real environment, have greater ecological validity, there were limitations to our study, including difficulties in controlling external variables. Despite this limitation, we believe that the present experiments succeeded in revealing some phenomena related to music listening while walking.

### Conflict of interest statement

The authors declare that the research was conducted in the absence of any commercial or financial relationships that could be construed as a potential conflict of interest.
