# Supplementary material for: Tempo and walking speed with music in the urban context
Source: Front Psychol. 2014 Dec 2;5:1361. doi: 10.3389/fpsyg.2014.01361 (PMC4251309; doi:10.3389/fpsyg.2014.01361)
Supplement: Supplementary file 1 [file DataSheet1.DOCX]

***Supplementary Material***

**Tempo and walking speed with music in the urban context**

**Marek Franěk_1_^1^*, Leon van Noorden_2_^2^, Lukáš Režný_3_^1^**

^1^Faculty of Informatics and Management, University of Hradec Králové, Hradec Králové, Czech Republic

^2^Institute for Psychoacoustics and Electronic Music, Department of Musicology, Ghent University, Ghent, Belgium.

*** Correspondence:** Marek Franěk, Faculty of Informatics and Management, University of Hradec Králové, Rokitanského 62, Hradec Králové, CZ 500 03, Czech Republic.

marek.franekl@uhk.cz

**Synchronization with music in Experiment 1**


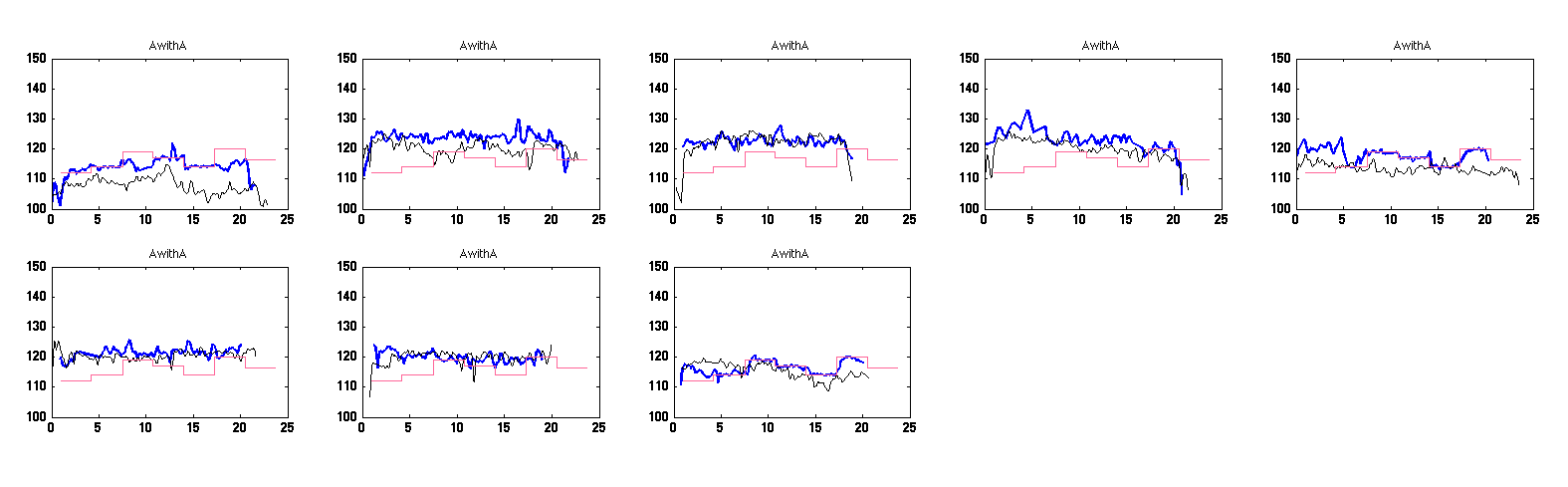
Synchronization between the tempo of musical pieces and the tempo of walking speed in Experiment 1. The data are plotted separately for each subject participating in the no-music condition and the music condition.

Supplementary Figure 1. The data for the participants walking in direction A while listening Music A. Time (in seconds) is plotted on x axis, tempo (bpm) on y axis. The black line represents the walking speed in the no-music condition. The blue line represents the walking speed while listening Music A in the music condition. The red lines show the speeds of particular pieces from the musical track. There were seven pieces in the musical track.

**
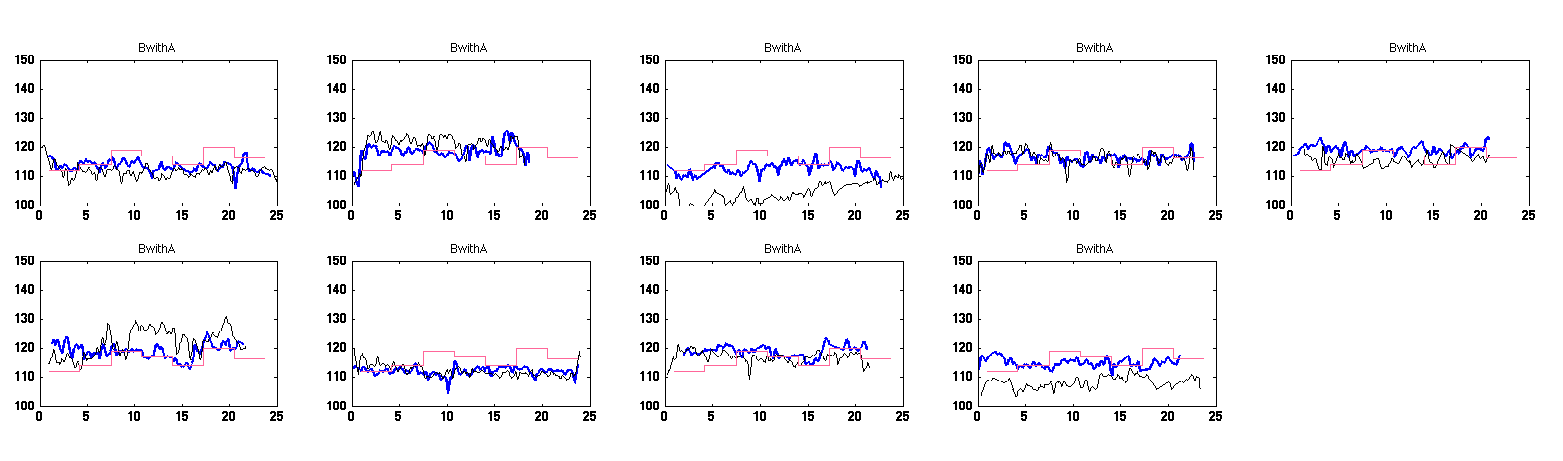
**

**Supplementary Figure 2. The data for the participants walking in direction B while listening Music A.** For details see the legend in Figure 1.

**
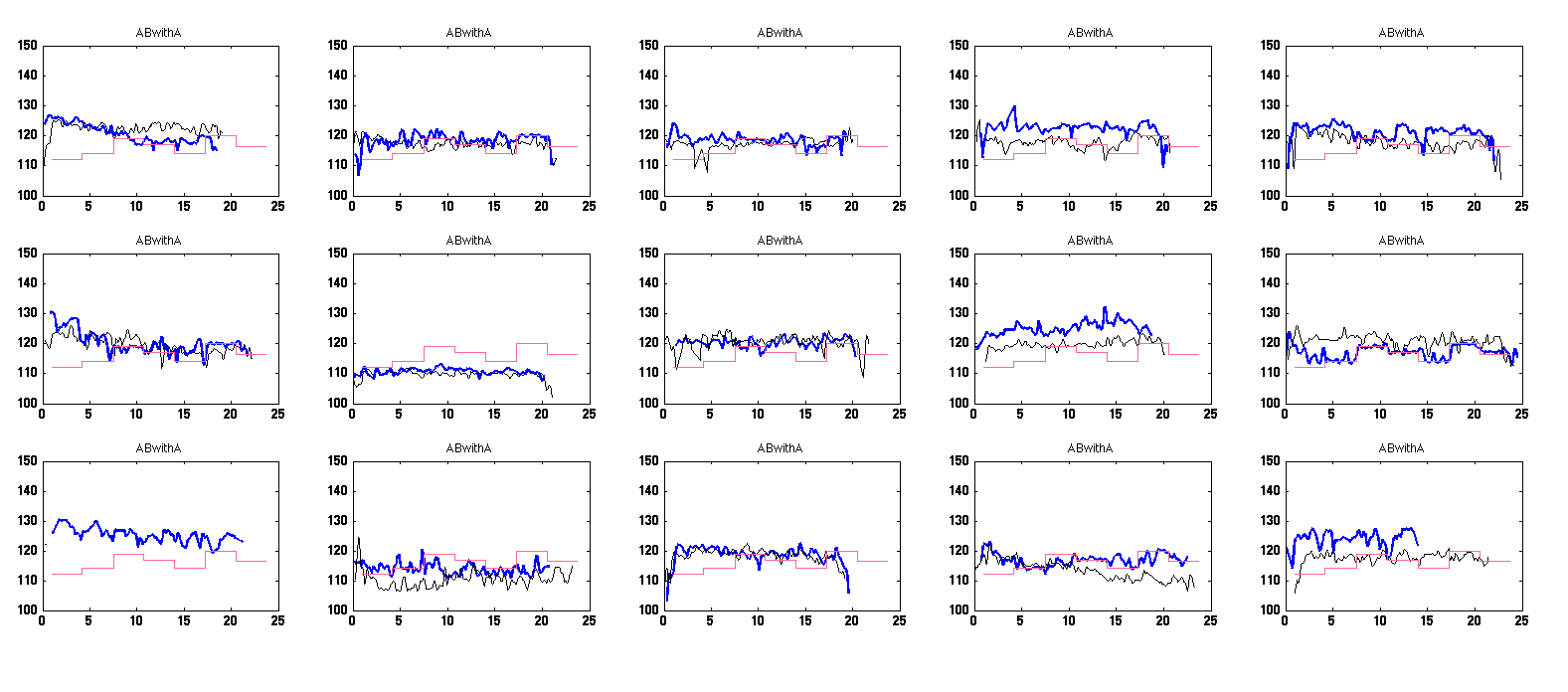
**

**Supplementary Figure 3. The data for the participants walking in direction A and B while listening Music A.** The participants walked in the two walks in different directions: in the no-music condition in the direction A and in the music condition in the direction B, and they had Music A in the second walk (and no music in the first walk). For details see the legend in Figure 1.

**
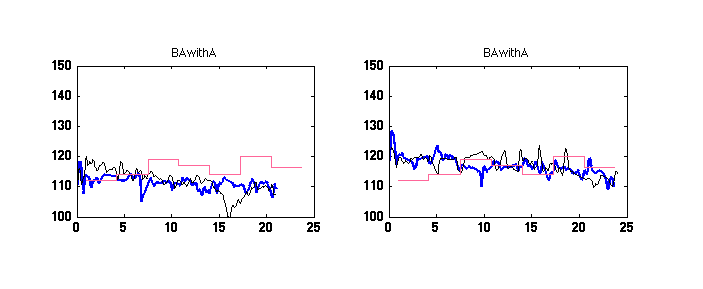
**

**Supplementary Figure 4. The data for the participants walking in direction B and A while listening Music A.** The participants walked in the two walks in different directions: in the no-music condition in the direction B and in the music condition in the direction A, and they had Music A in the second walk (and no music in the first walk). For details see the legend in Figure 1.

**
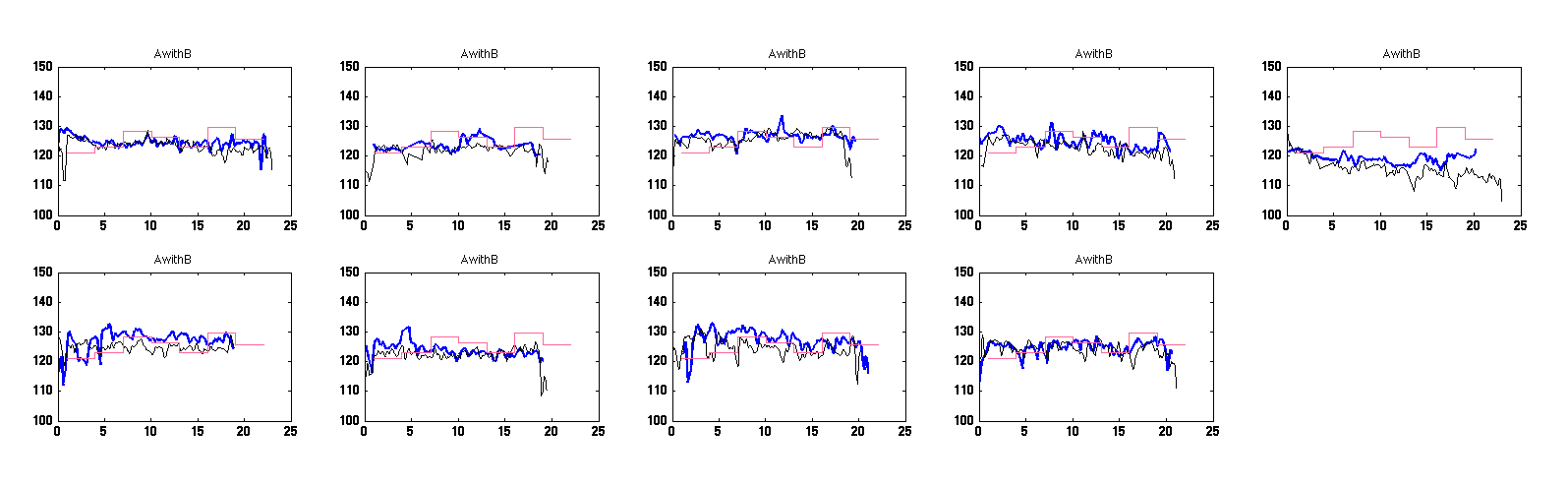
**

**Supplementary Figure 5. The data for the participants walking in direction A while listening Music B.** For details see the legend in Figure 1.

**
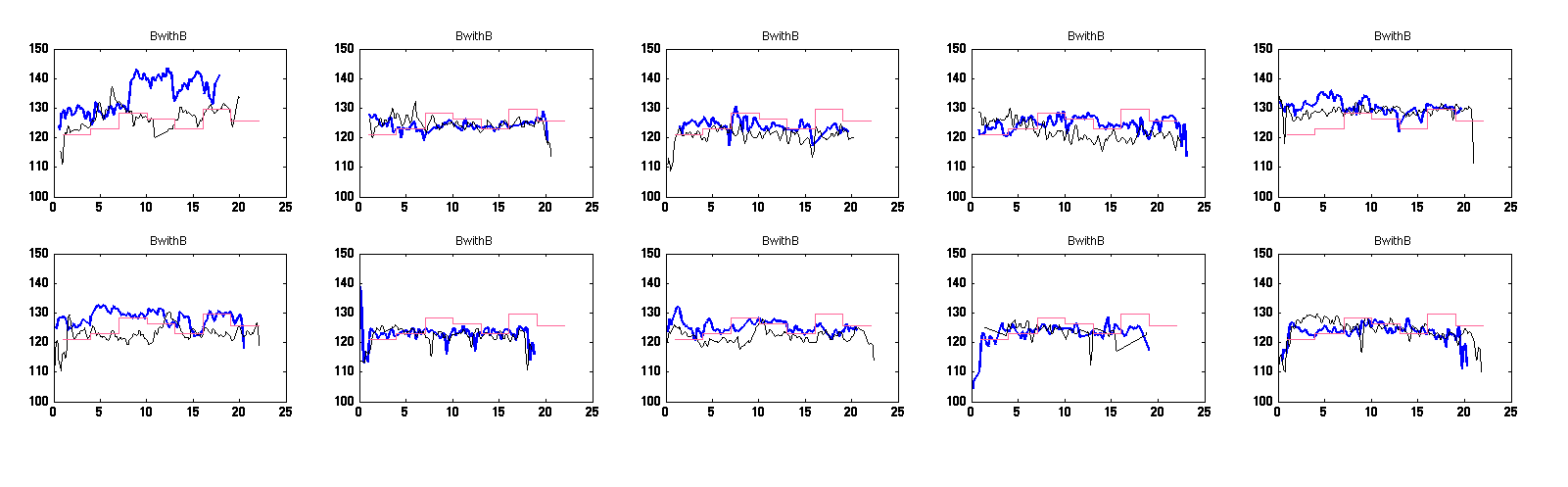
Supplementary Figure 6. The data for the participants walking in direction B while listening Music B.** For details see the legend in Figure 1.

**
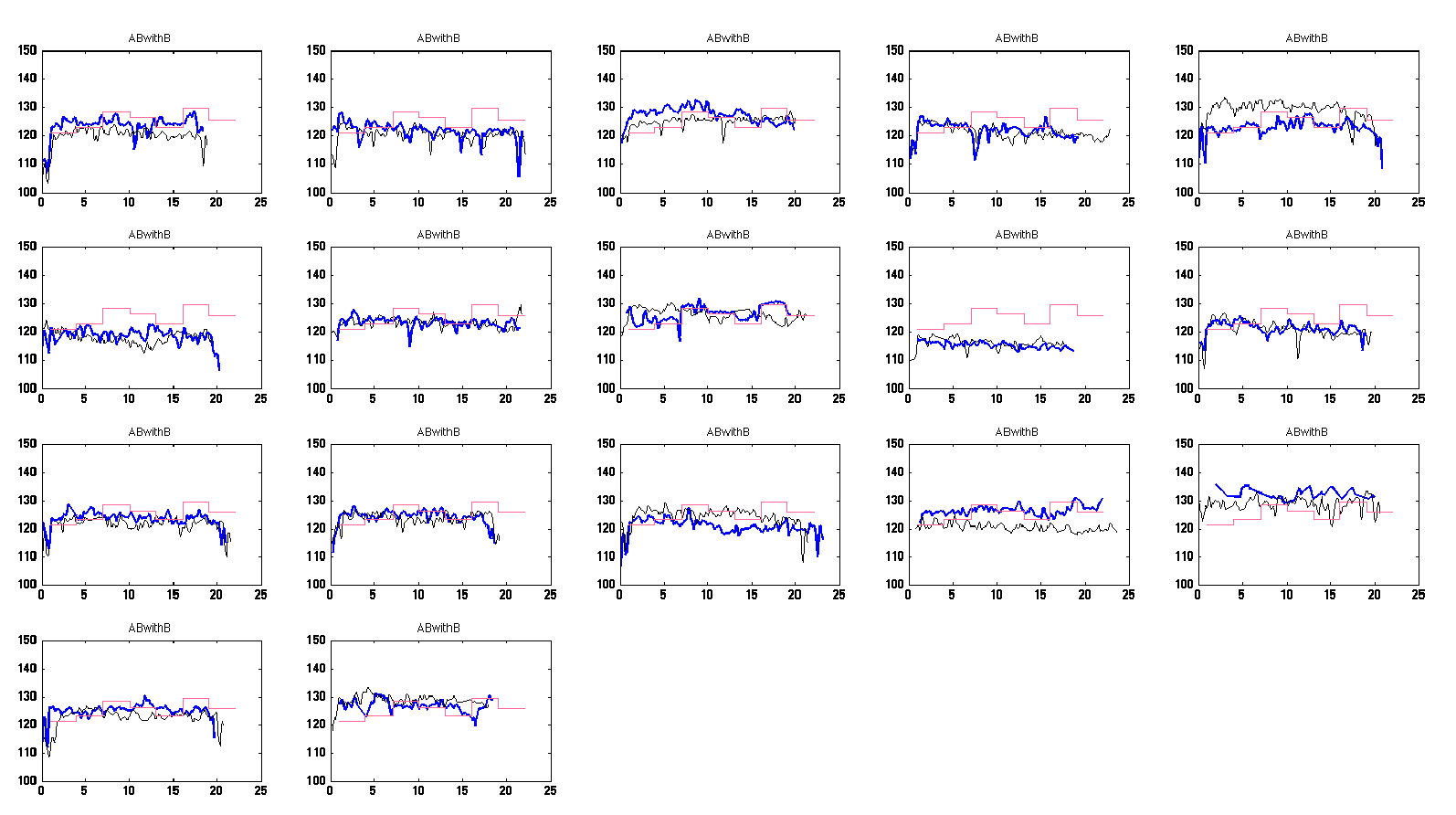
**

**Supplementary Figure 7. The data for the participants walking in direction A and B while listening Music B.** The participants walked in the two walks in different directions: in the no-music condition in the direction A and in the music condition in the direction B, and they had Music B in the second walk (and no music in the first walk).
